# Burn-injured skin is marked by a prolonged local acute inflammatory response of innate immune cells and pro-inflammatory cytokines

**DOI:** 10.3389/fimmu.2022.1034420

**Published:** 2022-11-14

**Authors:** Patrick P.G. Mulder, Marcel Vlig, Esther Fasse, Matthea M. Stoop, Anouk Pijpe, Paul P.M. van Zuijlen, Irma Joosten, Bouke K.H.L. Boekema, Hans J.P.M. Koenen

**Affiliations:** ^1^ Preclinical & Clinical Research, Association of Dutch Burn Centres (ADBC), Beverwijk, Netherlands; ^2^ Laboratory of Medical Immunology, Department of Laboratory Medicine, Radboud University Medical Center, Nijmegen, Netherlands; ^3^ Burn Center & Department of Plastic and Reconstructive Surgery, Red Cross Hospital, Beverwijk, Netherlands; ^4^ Department of Plastic Reconstructive and Hand Surgery, Amsterdam UMC Vrije Universiteit Amsterdam, Amsterdam, Netherlands; ^5^ Amsterdam Movement Sciences (AMS) Institute, Amsterdam UMC, Amsterdam, Netherlands; ^6^ Paediatric Surgical Centre, Emma Children’s Hospital, Amsterdam UMC University of Amsterdam, Amsterdam, Netherlands

**Keywords:** cell isolation, immune cells, flow cytometry, neutrophils, lymphocytes, macrophages, inflammation, burn wound tissue

## Abstract

The systemic and local immune response in burn patients is often extreme and derailed. As excessive inflammation can damage healthy tissues and slow down the healing process, modulation of inflammatory responses could limit complications and improve recovery. Due to its complexity, more detailed information on the immune effects of thermal injury is needed to improve patient outcomes. We therefore characterized and quantified subsets of immune cells and mediators present in human burn wound tissue (eschar), sampled at various time points. This study shows that after burn injury, the number of immune cells were persistently increased, unlike the normal wound healing process. There was an immediate, strong increase in neutrophils and a moderate increase in monocytes/macrophages and lymphocytes, especially in the second and third week post burn. The percentage of classical (CD14^high^CD16^-^) monocytes/macrophages demonstrated a steady decrease over time, whereas the proportion of intermediate (CD14^high^CD16^+^) monocytes/macrophages slowly increased. The absolute numbers of T cells, NK cells and B cells increased up to week 3, while the fraction of γδ T cells was increased only in week 1. Secretome profiling revealed high levels of chemokines and an overall pro-inflammatory cytokine milieu in burn tissue. The local burn immune response shows similarities to the systemic immune reaction, but differs in neutrophil maturity and lymphocyte composition. Altogether, the neutrophil surges, high levels of pro-inflammatory cytokines and limited immunosuppression might be key factors that prolong the inflammation phase and delay the wound healing process in burns.

## Graphical Abstract

Graphical overview of the study. Top part shows the investigation of burn wound tissue using flow cytometry, immunohistochemistry and cytokine analysis. Bottom part shows the relative response of immune cells during normal wound healing (as described in literature) and after burn injury (as shown by the study results) (1).

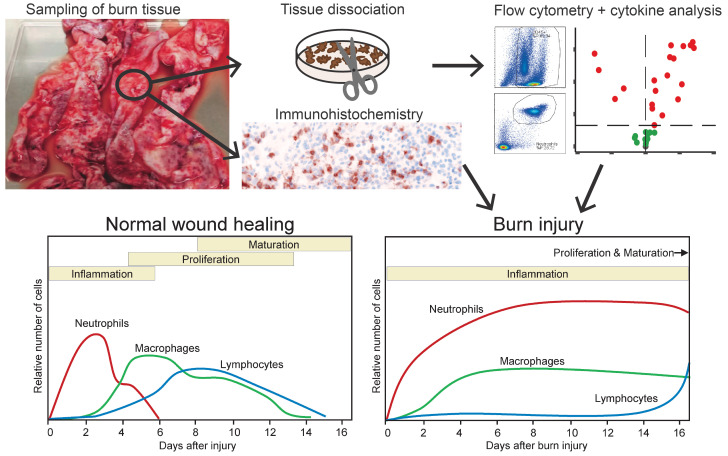

## Introduction

Burn injury is often accompanied by an extensive, derailed immune response in both burn wound tissue and peripheral blood (2, 3). Regardless of infection, burn patients generally show signs of systemic inflammation caused by high levels of cytokines and danger signals that originate from damaged tissue (4, 5). Necrotized and inflamed tissue stimulates the immune system to recruit acute phase immune cells to the affected site (3, 6, 7). Fibroblasts and keratinocytes surrounding the wound site and infiltrating leukocytes release a storm of cytokines, chemokines and growth factors that initiate the inflammation phase (8).

Typically during wound healing, neutrophils and macrophages with a pro-inflammatory (i.e. M1) phenotype will migrate into the wounded skin to remove debris and prevent bacterial colonization (9). Within days, wound neutrophils will disappear through apoptosis and macrophages will differentiate into a state that supports wound healing (i.e. M2 phenotype) (10). Generally within week after injury, lymphocytes will infiltrate the wound site to orchestrate tailored pathogen-eliminating and immune cell regulating responses (11). The reduction, transition and control of immune cells are crucial for dampening of the inflammatory response and for the establishment of a healthy wound healing process. After burn injury however, the immune system can be overactive and is then likely to cause damage to surrounding tissues, delay wound healing and contribute to the severity of scarring (3, 7).

Burn patients who experience persistent inflammation might benefit from immune suppressive treatment, however at the same time they are at risk of contracting infections such as pneumonia or cellulitis, caused by opportunistic bacteria (12). Therefore, innovative and precise interventions that modulate the immune response could be crucial in the relief of secondary illnesses while improving wound healing and preventing infection. Still, there is only little information on the immune response after burn injury and how exactly it differs from normal wound healing, mainly due to its complexity and variation among cases (e.g. burn size, depth and cause) and burn patients (e.g. age, sex and co-morbidities) (13). Moreover, present evidence on the processes that underlie burn injury originates mostly from animal research (14), which is only partially translatable to the human situation (15). We previously showed that in blood from severely burned patients, there was an extreme increase in innate immune cells and pro-inflammatory cytokines (8). In this longitudinal study, we investigated immune cells and soluble factors present in burn wound tissue (eschar) that was surgically debrided as part of standard treatment (16). A better understanding of the immune reactions to burn injury will facilitate the design of improved and more targeted treatment approaches for trauma-induced immune dysfunction.

## Materials and methods

### Sample collection

Burn wound tissue (eschar) from patients of all ages and thermal burn causes who underwent eschar debridement as part of their treatment at the Burn Center of the Red Cross Hospital in Beverwijk, the Netherlands. Healthy skin samples were obtained from adult patients who underwent cosmetic surgery (abdominoplasty or elective) at the Department of Plastic and Reconstructive Surgery of the Red Cross Hospital. Tissue samples were collected in the period between February 2019 and December 2021. Consent for the use of residual samples was received through the opt-out protocol of the Red Cross Hospital, in accordance with the national guidelines (https://www.coreon.org/). Subjects were informed of this procedure and were able to withdraw at any point. After surgical removal, tissue samples were stored in RPMI 1640 (Gibco, Paisley, UK) containing 1% penicillin and streptomycin (Gibco) as soon as possible to increase cell survival (17, 18). Samples were stored overnight at 4 °C and processed the following morning. Subject and sample characteristics are shown in **Supplementary Table 1**.

### Single cell isolation

This protocol was based on the immune cell isolation procedure from He et al. (19). Biopsies were taken from viable areas of the burn tissues, i.e. white or red areas with bleeding spots and not blackened or leathery areas. Approximately 600 mg of tissue was used per cell isolation for flow cytometry (FCM). Tissue samples were cut into smaller pieces and subsequently divided over 2 C-tubes (Miltenyi Biotec GmbH, Bergisch Gladbach, Germany) containing 5 mL of RPMI 1640 containing 1% penicillin and streptomycin. C-tubes were placed on a tissue dissociator (gentleMACS, Miltenyi Biotec) and program “B” was run. Hundred-fifty µL of 80 mg/mL collagenase I (Merck, St. Louis, MO, USA) in PBS (Gibco) was added and the sample was incubated for 1 h in a shaking water bath at 37 °C. After incubation, the C-tube was placed on the tissue dissociator to run program “B”. Samples were passed through a 500 µm and 40 µm cell strainer (pluriSelect, Leipzig, Germany) to obtain a single cell suspension. Suspensions were centrifuged for 10 min at 450 × *g*, and supernatant was discarded. The cell pellet was resuspended in erythrocyte lysis buffer (1.5 mM NH_4_Cl, 0.1 mM NaHCO_3_ and 0.01 mM EDTA in demineralized water) for 10 min at room temperature. Twenty mL of FCM buffer (PBS containing 1% BSA, 0.05% natrium-azide and 1 mM EDTA) was added and the suspension was centrifuged for 10 min at 450 × *g*. The pellet was resuspended in 5 mL of FACS buffer and cells were counted on the flow cytometer (MACS Quant Analyzer 10, Miltenyi Biotec GmbH, Bergisch Gladbach, Germany).

### Supervised flow cytometry

From the single cells suspensions approximately 2.5 × 10^5^ cells were used per staining panel. Antibodies used for FCM are displayed in **Supplementary Table 2**. A solution of 7-AAD (Miltenyi Biotec GmbH, Bergisch Gladbach, Germany) or propidium iodide (Miltenyi Biotec GmbH, Bergisch Gladbach, Germany) were used to calculate viability of cells. Stained cell samples were acquired on the MACS Quant Analyzer 10 and analysis was performed using FlowLogic (Inivai Technologies, Victoria, Australia). Gating strategy is shown in **Supplementary Figure 1**. Data was visualized using Graphpad version 5.01 (PRISM, La Jolla, USA) and R (ggplot package).

### Unsupervised flow cytometry analysis

Lymphocyte (panel 1), T cell (panel 2), neutrophil (panel 3) or monocytic (panel 4) populations were gated based on FSC/SSC, CD45, CD3, CD15 in MACSQuantify 2.13.3 software (Miltenyi Biotec). Data of these sole populations were uploaded to Cytobank (20) to create Flow Self-Organizing Map (FlowSOM) clusters.

### Immunohistochemistry

Kryofix (50% ethanol, 3% PEG300)-fixed paraffin-embedded 5 µm thick sections were used after deparaffinization and rehydration. Endogenous peroxidase was blocked in 1% hydrogen peroxide for 15 min at room temperature. Next, antigen retrieval for different antigens was performed. The blocking step was performed using 5% normal goat serum (Merck) diluted in PBS + 1% bovine serum albumin (BSA). Tissue sections were then incubated with the primary antibodies (**Supplementary Table 3**) for 1 h at RT followed by incubation with a poly-HRP-goat-anti-mouse or rabbit secondary antibody (BrightVision, VWR) for 30 min at RT. Detection of the target protein was established using 3,3′-Diaminobenzidine (BrightDAB, VWR). After immunohistochemical (IHC) DAB staining was successful, sections were counterstained with hematoxylin, dehydrated and mounted with Eukitt Mounting Medium (Merck). Percentage of MPO, CD3 or CD68 positive area was calculated using NIS Elements (Nikon Instruments Europe B.V.) and based on 3 images from representative tissue sections.

### Multiplex imaging and analysis

Formalin-fixed and paraffin-embedded 5 µm sections were deparaffinized using xylene and rehydrated with ethanol and distilled water. Antigen retrieval was performed by boiling in TRIS-borate-EDTA buffer for 10 min. A multiplex staining for the detection of neutrophils and lymphocytes was performed performed using the protocol described by Rodriguez et al. (21).

### Immunoassay of tissue homogenates

Frozen tissue samples of approximately 60 mg were thawed, minced into smaller pieces and further dissociated in M-tubes (Miltenyi Biotec) by adding lysis buffer (PBS containing 0.01 mM EDTA and protease inhibitor (1 tablet per 10 mL; Pierce, Thermo Scientific)) and running program “Protein_01” on a gentleMACS (Miltenyi Biotec). Debris was removed from the samples using a filter plate (Multiscreen, Merck) and samples were diluted to concentration of 12 mg tissue/mL. Luminex assay was performed according to the manufacturer’s instructions (Merck KGaA). The following assay kits were used: HCYTA-60K, TGFBMAG-64K, HCYP2MAG-62K and HTH17MAG-14K. In short, 25 µL of tissue homogenate was used to determine the concentrations of 37 soluble factors, namely MCP-1 (CCL2), MIP-1α (CCL3), MIP-1β (CCL4), MIP-3α (CCL20), GROα (CXCL1), IP-10 (CXCL10), IFN-α2, IFN-γ, TNF-α, TGF-β1, TGF-β2, TGF-β3, CTACK (CCL27), RANTES (CCL5), IL-1α, IL-1β, IL-2, IL-4, IL-5, IL-6, IL-8 (CXCL8), IL-9, IL-10, IL-12p40, IL-12p70, IL-13, IL-17A (CTLA-8), IL-17F, IL-18, IL-21, IL-22, IL-23, IL-33 (NF-HEV), GM-CSF, PDGF-AA, PDGF-AB/BB and VEGF-A. For TGF-β1,2,3 samples were acid-treated prior to the assay, according to the manufacturer’s instructions. Mean fluorescence intensity of samples was measured with a Flexmap 3D System (Luminex Corp, Austin, USA) and concentrations were calculated using Bio-Plex Manager Software (Bio-Rad Laboratories, Veenendaal, The Netherlands). Values below the minimum of the standard were based on extrapolation of the standard curve by the software.

### Statistical analyses

Distribution of the data was checked for normality using the Shapiro Wilk test. For the FCM and IHC data, differences between burn tissue and healthy skin, and between burn tissues of different time intervals after injury (PBW 1, 2, 3 and 4) were explored using the Mann Whitney U test in Graphpad version 5.01 (PRISM, La Jolla, USA). Only statistically significant differences are shown and are indicated by black asterisks (* = p < 0.05; ** = p < 0.01; *** = p < 0.001). The data was visualized using Graphpad version 5.01 (PRISM, La Jolla, USA). Levels of the soluble immune factors in burn tissue were transformed to fold change differences compared to the levels in healthy skin. P values of differences between burn tissue and healthy skin were determined using Mann Whitney U tests. We considered a p value of < 0.05 to be statistically significant. Volcano plots were created using “EnhancedVolcano” version 1.6.0 package in R version 3.6.2.

## Results

### Burn injury is followed by a strong local increase in granulocytes and moderate increase in monocytes and lymphocytes

**Figure 1:**
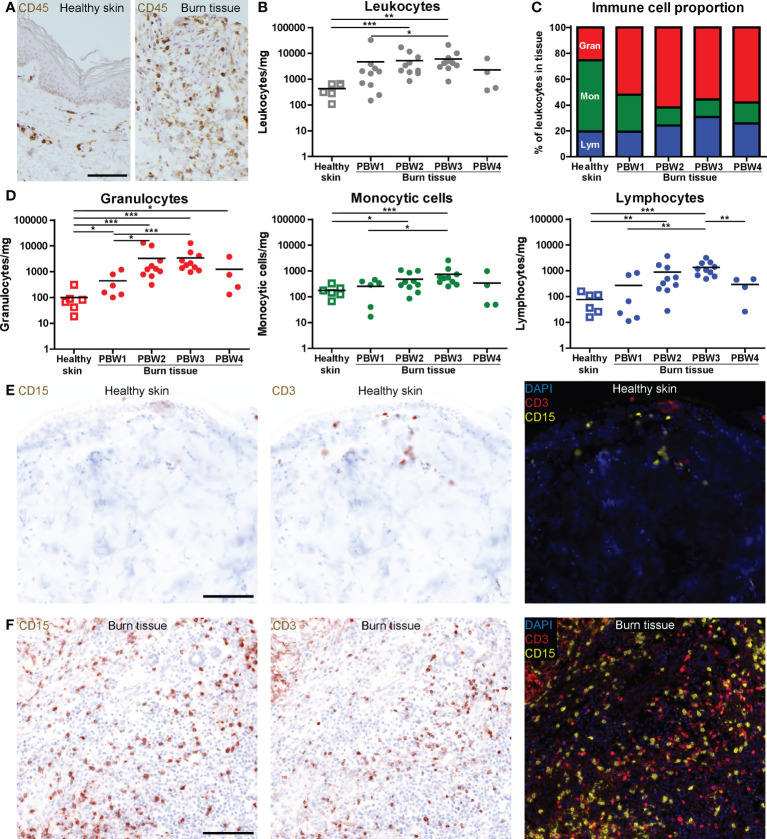
High number of immune cells infiltrate the skin as response to burn injury. **(A)** CD45 immunohistochemical DAB staining of a representative section of healthy skin and burn tissue (from 15 days post burn) (black scale bar = 100 µm). Flow cytometry-based quantification of: **(B)** Absolute number of leukocytes per mg tissue (based on side scatter and CD45); **(C)** Proportion of granulocytes (Gran), monocytic cells (Mon) and lymphocytes (Lym) in tissue (based on side scatter and CD45); **(D)** Absolute numbers of granulocytes, monocytic cells and lymphocytes per mg tissue (based on side scatter and CD45). Microscopic image of multiplex DAPI, CD15 (granulocytes) and CD3 (T cells) staining of a representative: **(E)** Healthy skin sample; **(F)** Burn tissue sample (from 25 days post burn), shown separately and as composite (black scale bar = 100 µm). P values were calculated using Mann-Whitney U statistical tests, significant differences are indicated by black asterisks: *p < 0.05; **p < 0.01; ***p < 0.001.

Local immune effects of burn trauma were investigated in burn tissue that was debrided during routine surgical procedures (subject and sample characteristics shown in **Supplementary Table 1**). We selected viable sections of tissue biopsies and neglected necrotized or blackened segments to ensure the isolation of viable cells. CD45 immunohistochemical (IHC) staining showed an extreme infiltration of leukocytes (CD45^+^ cells) in burn tissue (**Figure 1A**). The majority of leukocytes were viable after isolation from healthy skin (90.3% ± 6.6) and burn tissue (89.1% ± 10.5) (**Supplementary Figure 2A**). Flow cytometry (FCM)-based quantification revealed that the increase in leukocyte numbers was most abundant at post burn week (PBW) 2-3 (**Figure 1B**). As a result, the percentage of CD45^-^ cells, which include fibroblasts, keratinocytes and endothelial cells, was lower in burn tissue from PBW 2-3 than in healthy skin (**Supplementary Figure 2B**). The leukocytes isolated from healthy skin consisted of approximately 25% granulocytes, 55% monocytic cells (monocytes and macrophages) and 20% lymphocytes (**Figure 1C**). In burn tissue from PBW 1, there were 52% granulocytes, while for the proportion of monocytic cells was 29%. The lymphocytes fraction in burn tissue was similar to healthy skin (19%). In burn tissue from PBW 2-4, the portion of granulocytes was still enlarged (55-62%), while the fraction of monocytic cells decreased further to 13-16% and the lymphocyte fraction increased (24-31%). During PBW 1-3, absolute number of granulocytes, monocytic cells and lymphocytes rose and declined only at PBW 4 (**Figure 1D**). Multiplex spatial phenotyping of healthy skin and burn tissue sections using CD3 and CD15 revealed dense areas populated with granulocytes and T cells in burn tissue (**Figures 1E, F**).

### Granulocytes in burn tissue consist mainly of activated mature neutrophils

**Figure 2:**
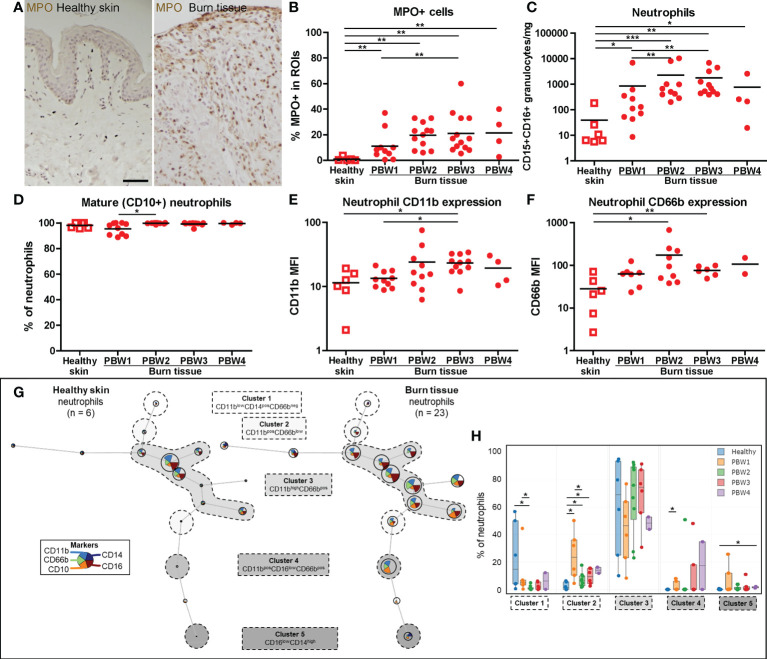
Local neutrophil response to burn injury. **(A)** Myeloperoxidase (MPO) immunohistochemical DAB staining of a representative section of healthy skin and burn tissue (from 15 days post burn) (black scale bar = 100 µm). **(B)** MPO^+^ area of tissue sections. Flow cytometry-based quantification of: **(C)** Absolute number of neutrophils (CD15^+^CD16^+^ granulocytes) per mg tissue; **(D)** Percentage of CD10^+^ (mature) neutrophils (CD15^+^CD16^+^ granulocytes); **(E)** MFI of CD11b on neutrophils (CD15^+^CD16^+^ granulocytes) in tissue; **(F)** MFI of CD66b on neutrophils (CD15^+^CD16^+^ granulocytes) in tissue. **(G)** Unsupervised clustering of neutrophil (CD15^+^CD16^+^ granulocytes) flow data from healthy skin and burn tissue, 5 clusters are highlighted. Node size represents relative size of population and node diagram shows expression level of markers. **(H)** Percentage of neutrophils within each cluster. Error bars in H show boxplot, p values were calculated using Mann-Whitney U statistical tests, significant differences are indicated by black asterisks: *p < 0.05; **p < 0.01; ***p < 0.001.

IHC analysis of myeloperoxidase (MPO) expression, an enzyme abundantly present in azurophilic granules of neutrophils (22), showed an immediate increase in neutrophil numbers in burn tissue at PBW 1, and an even larger increase from PBW 2 onward (**Figures 2A, B**). This was confirmed by FCM analysis of neutrophils (CD15^+^CD16^+^ granulocytes) (**Figure 2C**). Eosinophils (CD9^+^CD15^+^CD16^-^ granulocytes) were increased at PBW 2-3, but to a lesser extent (**Supplementary Figure 2C**). In both healthy skin and burn tissue neutrophils were almost exclusively CD10^+^, a marker that is associated with neutrophil maturation (23) (**Figure 2D**). Only in burn tissue from PBW 1 there was a slight increase in immature (CD10^-^) neutrophils. Activation markers CD11b and CD66b were upregulated in neutrophils at PBW 2-3 (**Figures 2E, F**). Self-organizing map clustering of flow data (FlowSOM) using Cytobank displayed cell populations (nodes) and clusters based on marker expression in an unsupervised manner (**Figure 2G**). This analysis highlights some of the burn-specific changes that occur in wound neutrophils. Burn injury caused significant differences in the percentage of neutrophils per cluster (**Figure 2H**). CD11b^low^CD14^+^CD66b^-^ neutrophils (cluster 1) were decreased early after burn injury, while CD11b^+^CD66b^low^ neutrophils (cluster 2) were increased. Although CD11b^high^CD66b^+^ neutrophils (cluster 3) seemed more represented in burn tissue than in healthy skin, no significant difference was found. A small population of CD16^low^ neutrophils (cluster 4) was significantly increased at PBW 1 and the percentage of CD16^low^CD14^+^ neutrophils (cluster 5) was significantly increased at PBW 4.

### Burn injury increases macrophage numbers and affects differentiation

**Figure 3:**
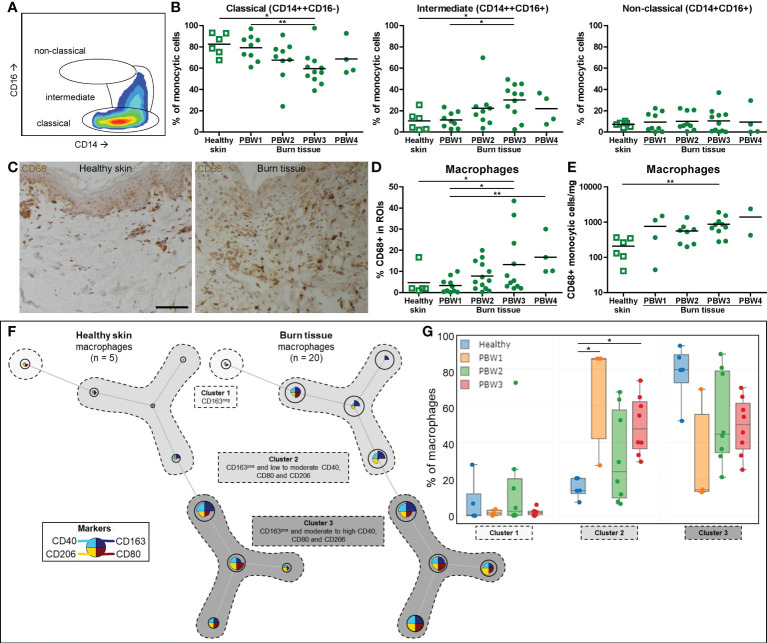
Local macrophage response to burn injury. **(A)** Flow cytometry gating strategy for detection of differentiation stages of monocytic cells (classical, intermediate or non-classical, as based on CD14 and CD16). **(B)** Flow cytometry-based quantification of percentage of monocytic cells within classical, intermediate, non-classical gates. **(C)** CD68 immunohistochemical DAB staining of a representative section of healthy skin and burn tissue (from 15 days post burn) (black scale bar = 100 µm). **(D)** CD68^+^ area of tissue sections. **(E)** Flow cytometry-based quantification of absolute number of macrophages (CD68^+^ monocytic cells) per mg tissue. **(F)** Unsupervised clustering of macrophages (CD68^+^ monocytic cells) in healthy skin and burn tissue, 3 clusters are highlighted. Node size represents relative size of population and node diagram shows expression level of markers. **(G)** Percentage of macrophages (CD68^+^ monocytic cells) within each cluster. Error bars in G show boxplot, p values were calculated using Mann-Whitney U statistical tests, significant differences are indicated by black asterisks: *p < 0.05; **p < 0.01.

Macrophage differentiation was assessed by analyzing the CD14 and CD16 expression of the monocytic cell population (among which are monocytes and macrophages) using flow cytometry. In both healthy skin and burn tissue the majority of these cells expressed a classical phenotype (CD14^high^CD16^-^) (**Figures 3A, B** **)**. In burn tissue from PBW 3, the proportion of classical monocytic cells decreased while the proportion of intermediate (CD14^high^CD16^+^) monocytic cells increased. Next, we analyzed the macrophages (CD68^+^ cells) within the monocytic cell population and found a steady increase in macrophages over time after burn injury (**Figures 3C–E** **)**. By both IHC and FCM we could detect a significant increase in macrophages at PBW 3. Macrophage phenotype was further investigated by analyzing CD40 and CD80 expression (indicative for pro-inflammatory phenotype) and CD163 and CD206 expression (hallmarks for pro-healing) (**Supplementary Figure 2D**). The only significant difference we observed was a reduction of CD40^+^ macrophages at PBW 3 (**Supplementary Figure 2D**). Using FlowSOM analysis of the FCM data, we identified macrophage subtypes with different expression patterns: CD163^-^ macrophages (cluster 1), CD163^+^ macrophages with a low or moderate expression of CD40, CD80 and CD206 (cluster 2) and CD163^+^ macrophages with a moderate to high expression of CD40, CD80 and CD206 (cluster 3) (**Figure 3F**). A significant increase in macrophages in cluster 2 was observed in burn tissue at PBW 1 and 3 (**Figure 3G**). Overall, this analysis demonstrated that burn injury increased the number of macrophages and changed their composition.

### Burn injury causes shifts in the lymphocyte composition and increases total T cells at PBW 2-3

**Figure 4:**
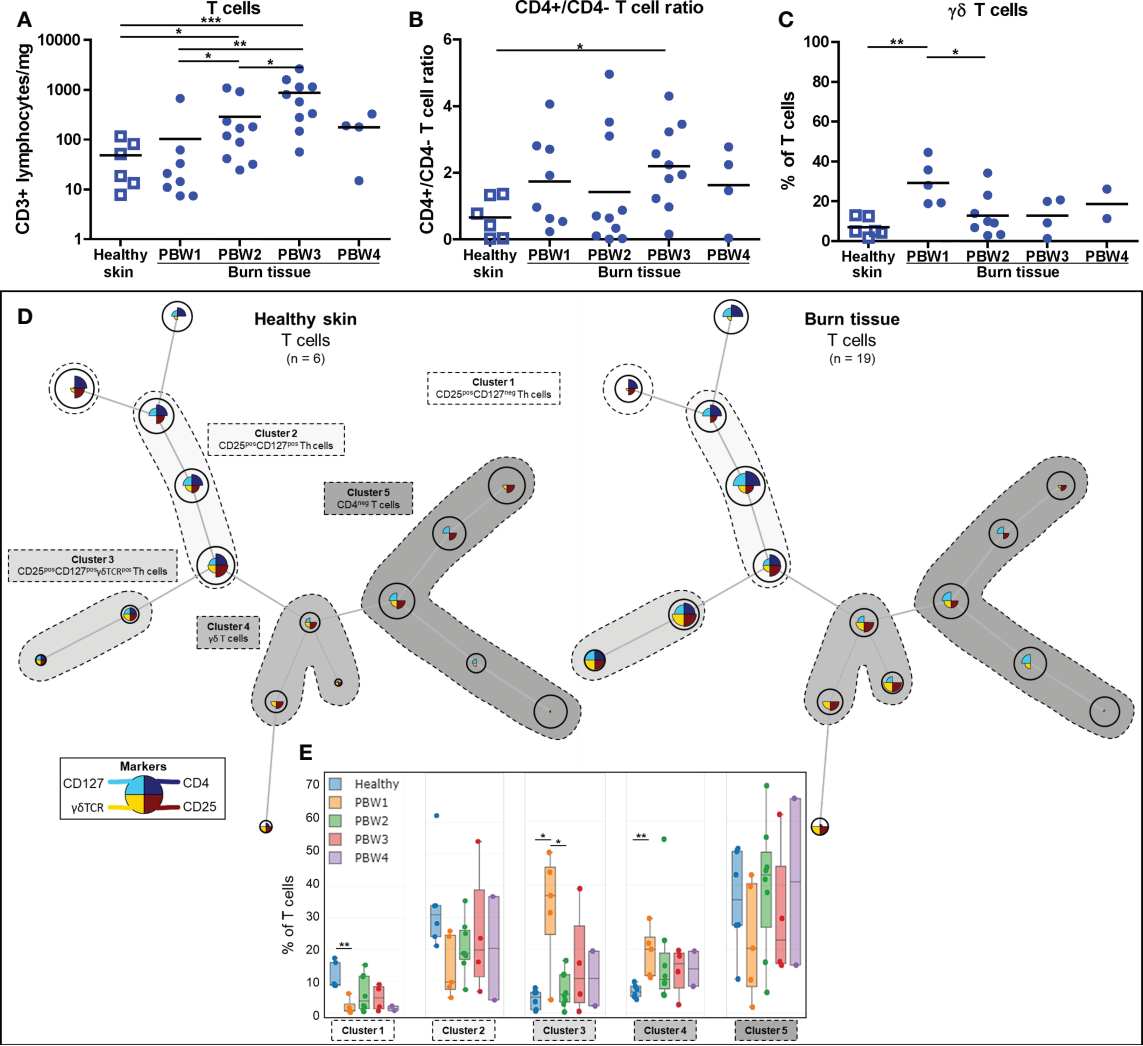
Local T cell response to burn injury. Flow cytometry-based quantification of: **(A)** Absolute number of T cells (CD3^+^ lymphocytes) per mg tissue; **(B)** CD4^+^/CD4^-^ T cell (CD3^+^ lymphocytes) ratio in tissue; **(C)** Percentage of T cells (CD3^+^ lymphocytes) that are γδ T cells (γδTCR^+^CD4^-^ T cells). **(D)** Unsupervised clustering of T cells (CD3^+^ lymphocytes) in healthy skin and burn tissue, 5 clusters are highlighted. Node size represents relative size of population and node diagram shows expression level of markers. **(E)** Percentage of T cells within each cluster. Error bars in E show boxplot, p values were calculated using Mann-Whitney U statistical tests, significant differences are indicated by black asterisks: *p < 0.05; **p < 0.01; ***p < 0.001.

T cell (CD3^+^ lymphocyte) numbers rose significantly at PBW 2-3 (**Figure 4A**), in line with the total lymphocyte increase (**Figure 1D**). A shift towards more CD4^+^ T cells was detected in burn tissue compared to healthy skin and were highest in burn tissue from PBW 3 as the CD4/CD8 T cell ratio (CD3^+^CD4^+^/CD3^+^CD4^-^ ratio) was higher in burn tissue than in healthy skin (**Figure 4B** and **Supplementary Figure 2E**). An increase in the proportion of γδ T cells (CD3^+^CD4^-^γδTCR^+^ lymphocytes) was found only at PBW 1 (**Figure 4C**), indicating a fast response of γδ T cells after burn injury. The absolute number of γδ T cells steadily increased over time after burn injury (**Supplementary Figure 2F**). The shift towards a higher abundance of γδ T cells at PBW 1 was confirmed by mapping flow cytometry data of T cells using FlowSOM (clusters 3 and 4; **Figures 4D, E** **)** and shows that the majority of the γδ T cells was CD25^+^, which is a prominent marker for cellular activation (24). At PBW 1 there was a relative decrease of T cells with a regulatory phenotype (CD25^+^CD127^-^; cluster 1). We did not observe considerable alterations in the cluster containing CD3^+^CD4^-^ T cells (cluster 5).

### Absolute number of NK and B cells increase after burn injury

**Figure 5:**
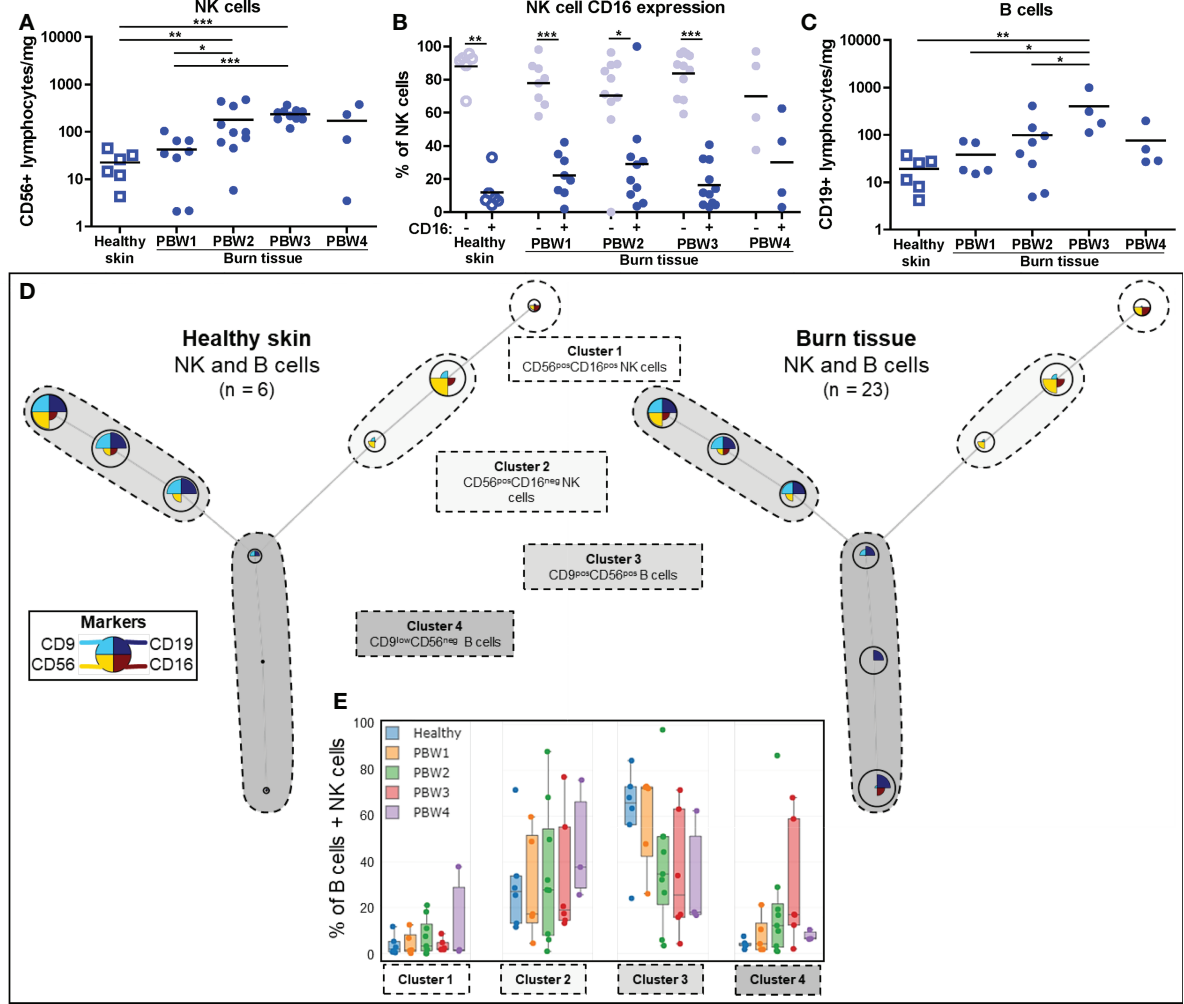
Local NK and B cell response to burn injury. Flow cytometry-based quantification of: **(A)** Absolute number of NK cells (CD56^+^ lymphocytes) per mg tissue; **(B)** Percentage of NK cells that are CD16^-^ and CD16^+;^ **(C)** Absolute number of B cells (CD19^+^ lymphocytes) per mg tissue. **(D)** Unsupervised clustering of NK and B cells in healthy skin and burn tissue, 4 clusters are highlighted. Node size represents relative size of population and node diagram shows expression level of markers. **(E)** Percentage of NK or B cells within each cluster. Error bars in E show boxplot, p values were calculated using Mann-Whitney U statistical tests, significant differences are indicated by black asterisks: *p < 0.05; **p < 0.01; ***p < 0.001.

FCM analysis showed that the absolute number of NK cells (CD56^+^ lymphocytes) was higher in burn tissue from PBW 2-3 (**Figure 5A**). Relative to total leukocyte numbers, NK cells were significantly reduced in burn tissue from PBW 1 and normalized afterwards (**Supplementary Figure 2F**). In both healthy skin and burn tissue, the majority of the NK cells was CD16^-^ (**Figure 5B**), which is opposed to the NK cell composition in peripheral blood where approximately 90% of the NK cells are CD16^+^ (25). Differences in CD16 expression of the NK cells were not observed between healthy skin and burn tissue or between time points. The absolute number of B cells (CD19^+^ lymphocytes) were higher in burn tissue from PBW 3 (**Figure 5C**), while the proportion of B cells within the leukocyte population in burn tissue was similar to that of healthy skin (**Supplementary Figure 2G**). We identified 4 clusters using FlowSOM analysis of the FCM data: CD56^+^CD16^+^ NK cells, CD56^+^CD16^-^ NK cells, CD9^+^CD56^+^ B cells and CD9^low^CD56^-^ B cells (**Figure 5D**). Clustering analysis showed a clear shift towards more CD9^low^CD56^-^ B cells in burn tissue but no significant differences were detected over time (**Figure 5E**).

### Immune cell infiltration coincides with high levels of cytokines, chemokines and growth factors

**Figure 6:**
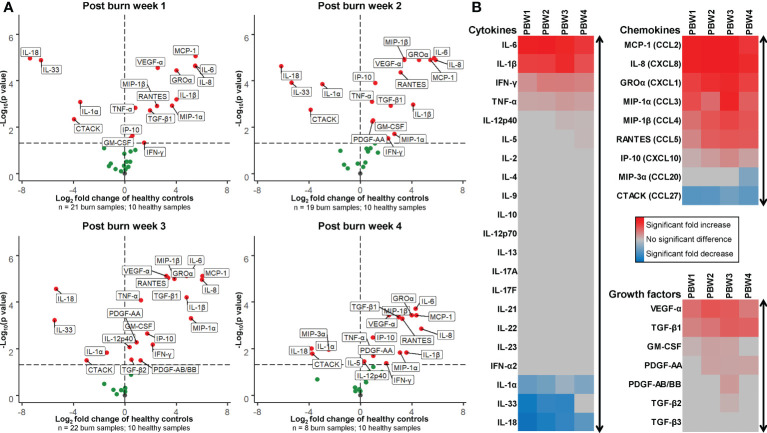
Expression of cytokines, chemokines and growth factors in burn tissue. **(A)** Volcano plot visualization of the expression of soluble factors in burn tissue from 1, 2, 3 and 4 weeks post burn injury. Dots represent soluble factors in burn tissue as Log_2_ fold change as found in healthy skin controls. Factors with a statistically significantly different expression (p < 0.05) are labeled (values above the black striped line). **(B)** Heatmap visualization of fold increase/decrease of soluble factors in burn tissue compared to healthy skin, categorized by cytokines, chemokines and growth factors. P values were calculated using Mann-Whitney U statistical tests.

The concentrations of 37 soluble immune factors were determined in homogenates of burn tissue using Luminex immunoassay (raw data is presented in **Supplementary Figure 3**). **Figure 6** shows an overview of these results using volcano plots and heatmaps at 4 time intervals after burn injury. In burn tissue there was an extremely high expression of IL-6, IL-1β, IFN-γ and TNF-α compared to healthy skin. The levels of these factors were persistently high, but for IL-6 and IFN- γ the levels declined at the later time intervals. Interestingly, increased levels of IL-12p40 and IL-5 were found only late after burn injury (PBW 3-4). As compared to healthy skin, a decrease was found for IL-1 family members IL-1α, IL-33 and IL-18. This is opposed to the level of IL-1β, which is also an IL-1 cytokine. The levels of IL-1α, IL-33 and IL-18 somewhat normalized at PBW 4 to the levels found in healthy skin. Chemokines MCP-1, IL-8, GROα, MIP-1α, MIP-1β, RANTES, IP-10 in burn tissue were increased at all analyzed time intervals, while the levels of T cell attracting chemokines CTACK and MIP-3α were decreased at PBW 1-4 and PBW 4, respectively. Among the growth factors, an increase in VEGF-A and TGF-β1 levels was found at PBW 1-4. From the growth factors, the level of GM-CSF was increased found at PBW 1-3, PDGF-AA at PBW 2-4 and PDGF-AB/BB and TGF-β2 only at PBW 3.

## Discussion

Next to being a protective, physical barrier, the skin carries out immune surveillance to ensure early and effective defense mechanisms against external threats. Besides fibroblasts and keratinocytes, healthy skin is inhabited mainly by lymphocytes and antigen presenting cells that survey the skin and react to foreign structures and danger signals (26). Here, we aimed to provide detailed insight in the cellular and soluble immune response in burn injured skin during the first four weeks after injury. In this study, we showed that after burn injury, there is a fast, extensive and long-lasting increase in innate immune cells that is present even in burn tissue debrided 3 to 4 weeks after injury. Lymphocytes also rise in numbers, but mainly at PBW 2-4. In addition, the cytokine composition in these burn tissue samples is highly pro-inflammatory and likely continues the attraction and activation of immune cells. Excessive pro-inflammatory immune responses and a lack or delay of anti-inflammatory responses could complicate wound healing and patient recovery. Limitations of this study that should be addressed are minor differences in treatment between patients such as medication and timing of surgery that could have influenced the inflammatory response. In addition, the broad range in subject age, burn cause and TBSA could have increased variation in the responses.

In tissue samples from PBW 1, the proportion of γδ T cells was increased, indicating that γδ T cells could play a role during the early phase of burn-induced response. γδ T cells possess a unique T cell receptor and can, unlike αβ T cells, interact with antigens directly (27). They execute immune surveilling functions and react to damaged cell structures by producing cytokines and chemokines to recruit immune cells (28). Mouse studies have shown that γδ T cells regulate the infiltration of innate immune cells shortly after trauma (29, 30). Our data suggests that next to keratinocytes, fibroblasts, mast cells and platelets (31), γδ T cells could be important inducers of the inflammatory response in humans as well. Within the same timeframe (PBW 1), IL-1β, IL-6, IL-8 (CXCL8), MCP-1 (CCL2), and GRO-α (CXCL1) levels were highly augmented. Others have demonstrated that these cytokines are also elevated in burn wound exudate (32). These factors are known enhancers of the inflammatory response and attract neutrophils and monocytes/macrophages to wound site (33). On the contrary, levels of IL-1α, IL-18 and IL-33 in burn tissue were reduced, especially during at PBW 1. These IL-1 cytokine family members are constitutively produced by keratinocytes to maintain the immune surveillance aspect of the skin (34). Reduction of these factors is presumably caused by extreme loss of keratinocytes due to destruction of the epidermal layer by thermal injury. In burn tissue from PBW 2-4, the levels of IL-1α, IL-18 and IL-33 were returning to the levels in healthy skin, which may be related to the presence of keratinocytes closing the defect. Levels of cytokines, as well as microRNAs (35), could be potential biomarkers to predict disease progression or recovery (36).

The rapid neutrophil response to burn injury is presumably caused by the persisting levels of neutrophil attractants, such as IL-8, MCP-1 and GRO-α. This can also be observed in the circulation of burn patients, where high levels of neutrophils were accompanied by high levels of IL-8 and MCP1, especially early after injury (8). Other studies have also shown that burn tissue contains large numbers of neutrophils in both human (37) and animals (14, 38). The vast majority of neutrophils that infiltrated the wound area were mature, whereas, in peripheral blood from severely burned patients high numbers of immature neutrophils were detected (8). This release of immature neutrophils may well be a compensatory response by the bone marrow (39). Nucleus flexibility and chemotactic activity increases with neutrophil age and could explain the presence of mainly mature neutrophils in burn tissue (40). If only mature neutrophils are able to enter the wound site, immature neutrophils would be trapped in the circulation until they reach maturity. As immature neutrophils are proposedly more active and less predictable in reacting to danger signals (41), they are likely to enhance systemic inflammation, thereby delaying recovery. In burn tissue, we found only a small number of immature neutrophils and only at PBW 1, which could have been released from the blood circulation by capillary leakage caused by the burn injury. Expression of CD11b and CD66b was increased on neutrophils isolated from burn tissue. This highlights the inflammatory state of the infiltrating neutrophils as CD11b and CD66b are important for neutrophil activation, adhesion and migration to inflamed tissue (42, 43). The surges of active neutrophils in the wound could lead to an overproduction of products such as elastase, MPO and ROS which can (further) damage surrounding tissues and organs (44, 45).

Blood monocytes are progenitors of both pro-inflammatory macrophages and wound healing macrophages. Although there is little evidence in this respect, it has been suggested that classical monocytes could be predisposed progenitors to pro-inflammatory macrophages (46), while intermediate and non-classical monocytes are biased progenitors to wound healing macrophages (47, 48). The initial monocyte population in burn tissue consisted mainly of classical monocytes. The relative decrease in classical monocytes in PBW 3 could indicate a relevant shift towards more wound healing macrophages, which is assumed to happen during wound healing (9). In burn tissue, the number of macrophages was increased this population showed a different composition of M1 and M2 markers. CD163^+^ macrophages with low to moderate expression of CD40, CD80 and CD206 were more abundant in burn tissue. M1 macrophage differentiation factor GM-CSF was increased in burn tissue from PBW 1-3 and mediators that are known to be actively produced by M1 macrophages such as TNF-α, IFN-γ, IL-1β, IL-6, IL-8 and MCP-1 (CCL2) (9, 49), were all increased in these burn tissues. While typical M2 macrophage factors like IL-4, IL-10, IL-13 were unaffected, the levels of TGF-β1 and VEGF-α, which are also described as M2 mediators (48), were increased in burn tissue. Altogether, the monocyte/macrophage composition and cytokine environment possibly supports the generation of macrophages with a pro-inflammatory phenotype. Timely transition towards more suppressive, regenerative macrophages is however essential for a healthy healing process, as these cells support fibroblasts in the formation of collagen and enhance re-epithelization (9, 48). Due to the active, continuing inflammation and the presence of danger signals from tissue damage, macrophage transition might be delayed or insufficient, although more research is required to elucidate this.

Immunosuppression from the adaptive arm of the immune system is essential to create an environment in which fibroblasts and keratinocytes can repair the damaged skin (50). Here, we revealed that lymphocyte numbers, including T cells, NK cells and B cells, were increased at PBW 2-3, which is relatively late after injury (11). This coincided with a high levels of chemokines MIP-1α (CCL3), MIP-1 β (CCL4) and RANTES (CCL5), which are known to attract lymphocytes to injured skin (11). Particularly CCL3, CCL4 and CCL5 are involved in the activation of NK cells (51) and could lead to increased cytokine release by NK cells in burn tissue. Information on the response of NK cells and B cells in burn tissue is very limited at this moment. We here showed that after burn injury there is an increased number of NK cells and B cells in burn tissue, however, functional assays are needed in order to speculate about their behavior and involvement in the burn immune response. The levels of CCL3, CCL4, CCL5, IFN-γ, TNF-α and IP-10 (CXCL10) were associated with the number of T cells at PBW 3. IP-10 promotes chemotaxis and inflammation and is likely induced by IFN-γ. Peters et al. previously described an interplay of keratinocytes and T cells and showed that IP-10 is actively produced by keratinocytes in co-culture, even with relatively low numbers of keratinocytes (52). This interplay is presumably also active during burn wound healing by residual, surrounding or re-epithelializing keratinocytes. Cytokines with anti-inflammatory properties such as IL-4, IL-10 and IL-13 were not detected in these burn tissue samples. Altogether, the soluble factors in burn tissue are likely to support Th1 response, resulting in more attraction of leukocytes to the wound site, while control or suppression of inflammation appears to be limited.

In this study, we showed that after burn injury, the numbers of immune cells were persistently elevated, while during normal wound healing neutrophils disappear within days and lymphocytes counts start to increase in the first week (10, 53, 54). Burn injury often leads to a prolonged hyperinflammatory state (3, 55) and treatment of burn wounds is therefore a difficult and time-consuming process. Damage to the skin is a trigger for the immune system to recruit immune cells en masse and replenish these immune cells in the blood from the bone marrow. Ancillary damage and chemokine production by immune cells and stressed skin cells will trigger the immune system to react, thereby creating a vicious circle of prolonged inflammation in both the skin and in the blood. Therapy is often empiric due to the large diversity among patients and their injuries, e.g. burn type, size, depth and location. In the present study, there was no indication that burn size or burn cause (water versus flame) affected cellular or soluble inflammatory markers (data not shown). Excessive and persistent inflammation is also among the causes of long-term complications such as the formation of hypertrophic scars (7). On top of that, there is a risk of contracting an infection and the activity of the immune system is unpredictable. In clinical practice, patients with burns larger than 15% TBSA are hypermetabolic and often develop SIRS or organ insufficiency. Hence debridement of burn tissue is important to reduce inflammation and promote wound healing while also preventing further tissue necrosis and cellulitis. Possibly, early debridement of burn tissue (noted as post-burn days 2 through 12) or impediment of pro-inflammatory cytokines such as IL-6 might remove inflammatory triggers at an early stage and avoid secondary damage (56, 57). Resolution of excessive inflammation using immune suppressants could increase the patients’ recovery rate, but might increase the risk for infection. Moreover, it can be very difficult to discriminate burn-induced SIRS from sepsis. Our analysis of the local immune reactions to burn injury aids in improving our understanding of burn-induced inflammation. This knowledge is needed to design more sophisticated and effective ways to diagnose and treat immune dysfunction and hyperactive inflammation. Immune modulating treatment targeting the disturbed immune processes will improve patients’ overall health recovery time and scar quality.

In conclusion, through the characterization of immune cell subsets isolated from human burn tissue we demonstrated that burn injury induced a local persistent surge of pro-inflammatory immune cells and cytokines, while immunosuppression appeared to be limited. These burn-induced immune reactions might be key factors that extent the inflammation phase and thereby obstruct the wound healing process in burn injury.

## Data availability statement

The raw data supporting the conclusions of this article will be made available by the authors, without undue reservation.

## Ethics statement

Tissue samples were obtained from planned surgeries that were part of routine patient therapy. Data and material was only used after consent from subjects (or legal representatives) using the opt-out procedure of the Red Cross Hospital in Beverwijk. This procedure is in accordance with the national guidelines (https://www.coreon.org/) and institutional guidelines of the Red Cross Hospital in Beverwijk in the Netherlands. No animals were used in these experiments.

## Author contributions

Conceptualization: PM, MV, IJ, BB, and HK. Methodology: PM, MV, IJ, BB, HK, and AP. Investigation: PM, MV, and EF. Resources: MS, AP, and PVZ. Formal analysis: PM, MV, BB, and HK. Visualization: PM and MV. Supervision: IJ, BB, and HK. Writing – original draft: PM. Writing – review & editing: MV, EF, MS, AP, PVZ, IJ, BB, and HK. All authors have read and approved the submission of this manuscript.

## Funding

Dutch Burns Foundation awarded grant WO/17.108 to BB to execute this project.

## Acknowledgments

We would like to thank Xuehui He for helping with the cell isolation protocol, Bram van Cranenbroek for his assistance on the immunoassay, Mark Gorris for helping with the multiplex imaging and Evi Warmerdam, Rosa Rentenaar and Myrthe Witbaard for their technical assistance during experiments. We are grateful for the work and participation of all involved physicians, surgeons, nurses and patients of the Burn Center and the Department of Plastic and Reconstructive Surgery of the Red Cross Hospital.

## Conflict of interest

The authors declare that the research was conducted in the absence of any commercial or financial relationships that could be construed as a potential conflict of interest.

## Publisher’s note

All claims expressed in this article are solely those of the authors and do not necessarily represent those of their affiliated organizations, or those of the publisher, the editors and the reviewers. Any product that may be evaluated in this article, or claim that may be made by its manufacturer, is not guaranteed or endorsed by the publisher.

## Supplementary material

Supplementary Figure 1 Gating strategy of flow cytometric analysis. At the top of the plots the panel numbers are shown for which the gating was performed (P1, 2, 3 and 4).

Supplementary Figure 2 Cell counts in burn tissue. Flow cytometry-based quantification of: **(A)** Percentage of leukocytes that stained negative for 7-AAD or propidium iodide (viable cells); **(B)** Percentage of isolated cells that is CD45- (fibroblasts, keratinocytes, endothelial cells, others); **(C)** Number of eosinophils (CD9^+^CD16^-^ granulocytes) per mg tissue; **(D)** Percentage of macrophages that is positive for CD40, CD80, CD163 or CD206; **(E)** Percentage of T cells that are CD4^-^ and CD4^+^. **(F)** Number of γδ T cells (γδTCR^+^) per mg tissue; **(G)** Percentage of NK cells (CD56^+^ lymphocytes) within leukocyte population; **(H)** Percentage of B cells (CD19^+^ lymphocytes) within leukocyte population. P values were calculated using Mann-Whitney U statistical tests, significant differences are indicated by black asterisks: *p < 0.05; **p < 0.01; ***p < 0.001.

Supplementary Figure 3 Concentrations of soluble factors in burn tissue. Healthy skin was used as controls. Black lines show mean values and the black striped line represents the lowest limit of detection. P values were calculated using Mann-Whitney U statistical tests, significant differences are indicated by black asterisks: *p < 0.05; **p < 0.01; ***p < 0.001.
