# Supplementary figures and images for: Burn-injured skin is marked by a prolonged local acute inflammatory response of innate immune cells and pro-inflammatory cytokines
Source: Front Immunol. 2022 Nov 14;13:1034420. doi: 10.3389/fimmu.2022.1034420 (PMC9703075; doi:10.3389/fimmu.2022.1034420)

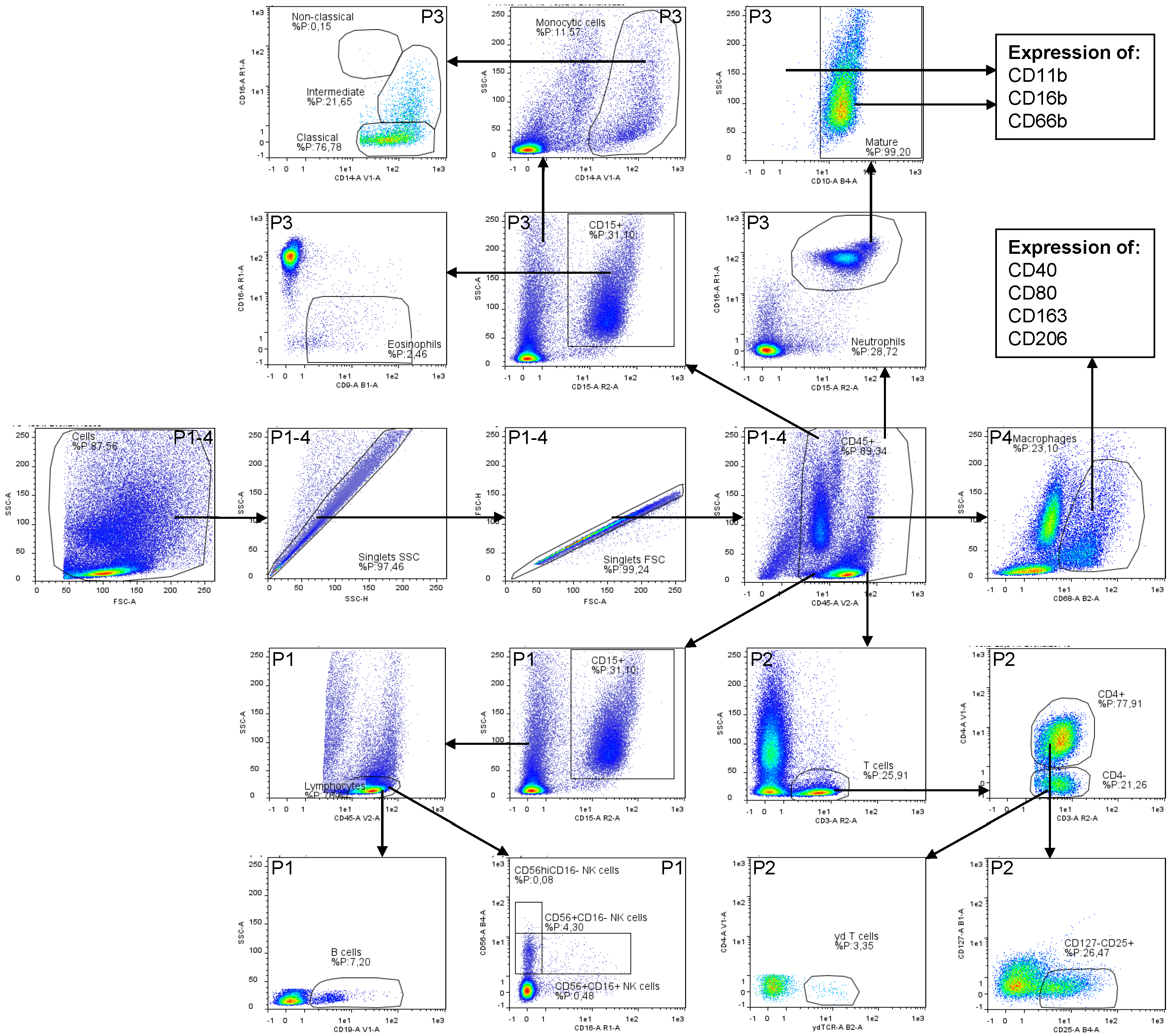

Supplement: Supplementary Figure 1 — Gating strategy of flow cytometric analysis. At the top of the plots the panel numbers are shown for which the gating was performed (P1, 2, 3 and 4). [file Image_1.tif]

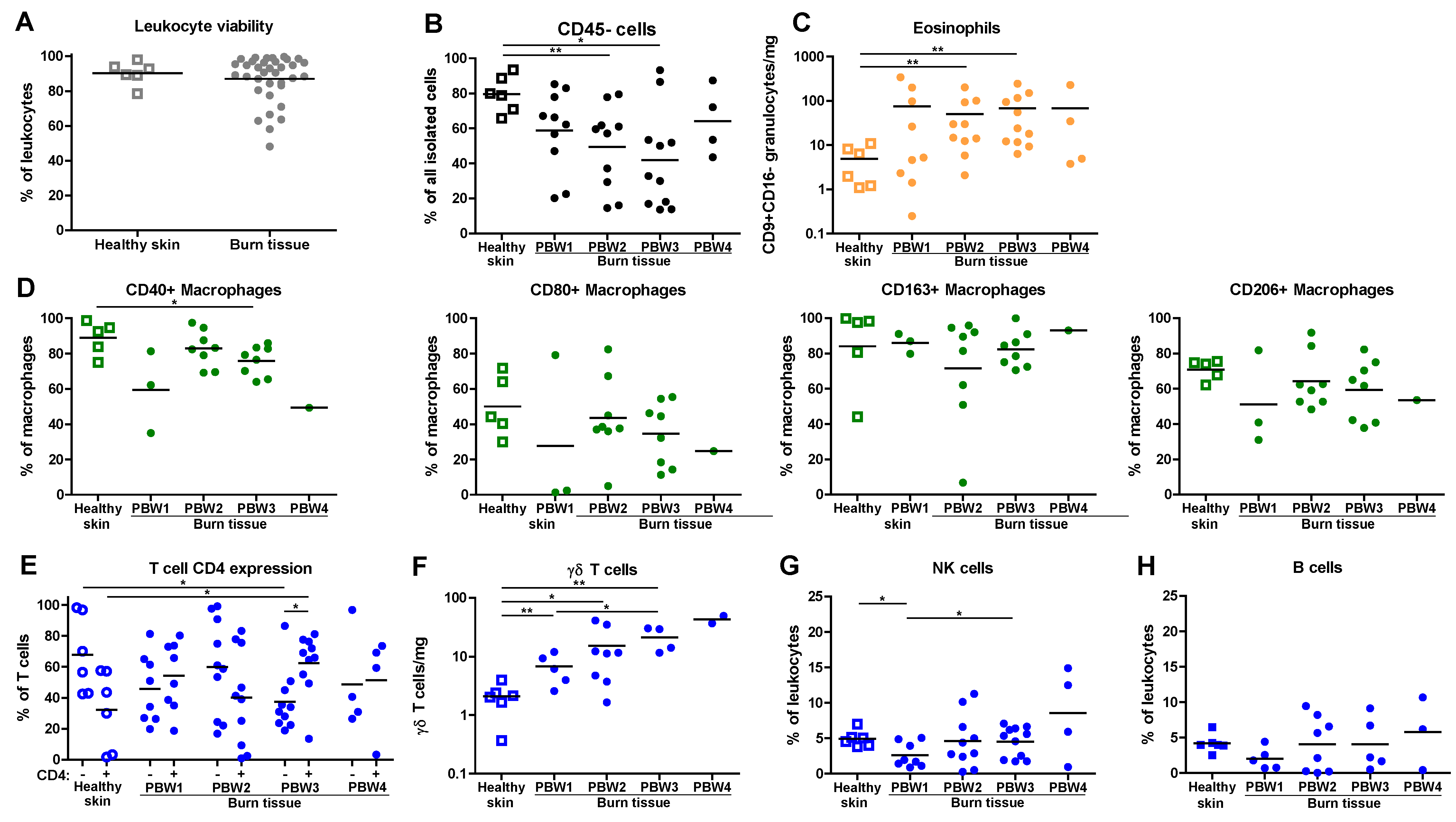

Supplement: Supplementary Figure 2 — Cell counts in burn tissue. Flow cytometry-based quantification of: (A) Percentage of leukocytes that stained negative for 7-AAD or propidium iodide (viable cells); (B) Percentage of isolated cells that is CD45- (fibroblasts, keratinocytes, endothelial cells, others); (C) Number of eosinophils (CD9+CD16- granulocytes) per mg tissue; (D) Percentage of macrophages that is positive for CD40, CD80, CD163 or CD206; (E) Percentage of T cells that are CD4- and CD4+. (F) Number of γδ T cells (γδTCR+) per mg tissue; (G) Percentage of NK cells (CD56+ lymphocytes) within leukocyte population; (H) Percentage of B cells (CD19+ lymphocytes) within leukocyte population. P values were calculated using Mann-Whitney U statistical tests, significant differences are indicated by black asterisks: *p < 0.05; **p < 0.01; ***p < 0.001. [file Image_2.tif]

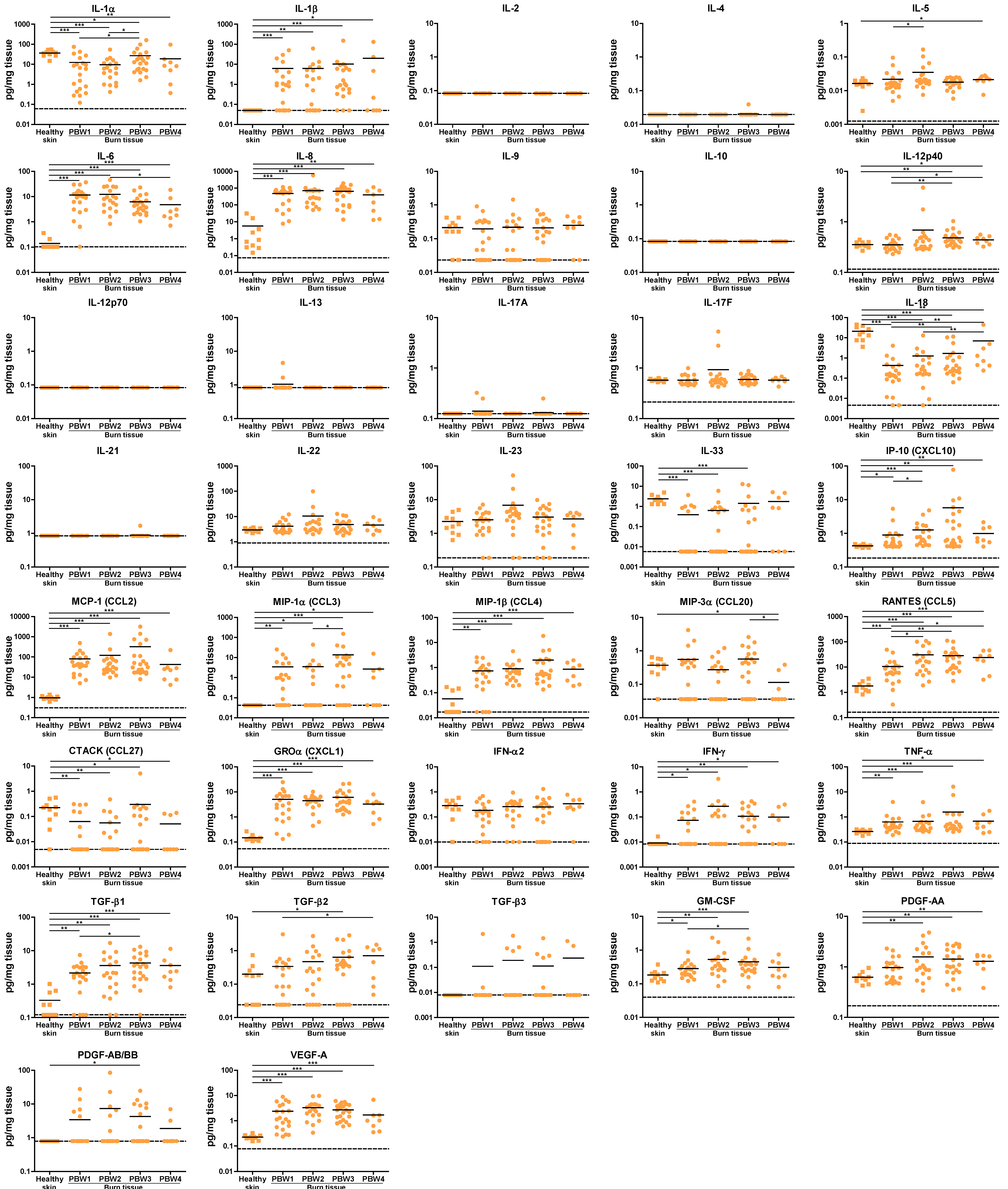

Supplement: Supplementary Figure 3 — Concentrations of soluble factors in burn tissue. Healthy skin was used as controls. Black lines show mean values and the black striped line represents the lowest limit of detection. P values were calculated using Mann-Whitney U statistical tests, significant differences are indicated by black asterisks: *p < 0.05; **p < 0.01; ***p < 0.001. [file Image_3.tif]
