# Supplementary material for: Burn-injured skin is marked by a prolonged local acute inflammatory response of innate immune cells and pro-inflammatory cytokines
Source: Front Immunol. 2022 Nov 14;13:1034420. doi: 10.3389/fimmu.2022.1034420 (PMC9703075; doi:10.3389/fimmu.2022.1034420)
Supplement: Supplementary file 4 [file Table_1.docx]

**SUPPLEMENTARY TABLES**

**Supplementary Table 1. Subject and sample characteristics.** Table shows number of samples, unless indicated otherwise. Inclusion criteria burn tissue: patients of all ages with thermal burn injuries treated who underwent eschar debridement as part of their treatment at the Burn Center of the Red Cross Hospital in Beverwijk, The Netherlands. Exclusion criteria burn tissue: freeze injuries, wounds with diagnosed infection, debridement using hydrosurgery system. All tissue samples were processed within 48 h.

|  |  | **Burn tissue** | **Healthy skin** |
| --- | --- | --- | --- |
| **Subject characteristics** | Samples | 83 | 20 |
|  | Subjects | 81 | 20 |
|  | Sex (M/F/unknown) | 44/35/2 | 2/17/1 |
|  | Age (mean ± SD) | 54 ± 19 y | 48 ± 12 y |
| **Anatomical location** | Arm | 20 | 1 |
|  | Leg | 32 | 1 |
|  | Torso | 11 | 12 |
|  | Multiple | 3 | 0 |
|  | Unknown | 17 | 6 |
| **Time after burn injury in weeks** | PBW 1 | 23 |  |
|  | PBW 2 | 22 |  |
|  | PBW 3 | 28 |  |
|  | PBW 4 | 10 |  |
| **Burn size** | TBSA (mean ± SD)^*^ | 17 ± 14% |  |
| **Burn cause** | Flame | 50 |  |
|  | Water | 18 |  |
|  | Oil/wax | 6 |  |
|  | Contact | 4 |  |
|  | Chemical | 2 |  |
|  | Electrical | 1 |  |

**^*^**Based on TBSA of 35 patients

**Supplementary Table 2. Antibodies used for flow cytometric analysis.**

| **Antibody** | **Clone** | **Conjugate** | **Manufacturer** |
| --- | --- | --- | --- |
| anti-CD3 | REA613 | APC | Miltenyi Biotec |
| anti-CD4 | REA623 | VioBlue | Miltenyi Biotec |
| anti-CD10 | REA877 | PE-Vio770 | Miltenyi Biotec |
| anti-CD11b | REA713 | FITC | Miltenyi Biotec |
| anti-CD14 | REA599 | VioBlue | Miltenyi Biotec |
| anti-CD15 | W6D3 | APC-fire750 | BioLegend |
| anti-CD16 | REA423 | APC | Miltenyi Biotec |
| anti-CD19 | REA675 | VioBlue | Miltenyi Biotec |
| anti-CD25 | REA945 | PE-Vio770 | Miltenyi Biotec |
| anti-CD40 | REA733 | FITC | Miltenyi Biotec |
| anti-CD45 | REA747 | VioGreen | Miltenyi Biotec |
| anti-CD56 | REA196 | PE-Vio770 | Miltenyi Biotec |
| anti-CD66b | REA306 | PE | Miltenyi Biotec |
| anti-CD68 | REA886 | APC-Vio770 | Miltenyi Biotec |
| anti-CD80 | REA661 | APC | Miltenyi Biotec |
| anti-CD127 | REA614 | FITC | Miltenyi Biotec |
| anti-CD163 | REA812 | PE | Miltenyi Biotec |
| anti-CD206 | DCN228 | PE-Vio770 | Miltenyi Biotec |
| anti-γδTCR | REA591 | PE | Miltenyi Biotec |

**Supplementary Table 3. Antibodies used for immunohistochemistry**

| **Antibody** | **Clone** | **Manufacturer** | **Dilution** | **Antigen retrieval** | **Stain** |
| --- | --- | --- | --- | --- | --- |
| anti-CD3 | Sp7 | ThermoFisher | 1/200 | EDTA | TSA520 |
| anti-CD3 | Sp7 | Abcam | 1/200 | EDTA | DAB |
| anti-CD8 | C8/144B | DAKO | 1/200 | EDTA | TSA690 |
| anti-CD15 | MMA | BD Biosciences | 1/400 | EDTA | TSA520/DAB |
| anti-CD45 | 2B11+PD7/26 | DAKO | 1/100 | Citrate | DAB |
| anti-CD68 | KP1 | DAKO | 1/2000 | EDTA | DAB |
| anti-MPO | Polyclonal | DAKO | 1/1200 | Citrate | DAB |
